# Shining Light on the Dark Side of the Genome

**DOI:** 10.3390/cells11030330

**Published:** 2022-01-19

**Authors:** Lori L. Wallrath, Felipe Rodriguez-Tirado, Pamela K. Geyer

**Affiliations:** Department of Biochemistry and Molecular Biology, Carver College of Medicine, University of Iowa, Iowa City, IA 52242, USA; felipe-rodriguez@uiowa.edu

**Keywords:** canalization, centromere, heat shock, heterochromatin, HSP90, piRNA, telomere, transposon, Waddington

## Abstract

Heterochromatin has historically been considered the dark side of the genome. In part, this reputation derives from its concentration near centromeres and telomeres, regions of the genome repressive to nuclear functions such as DNA replication and transcription. The repetitive nature of heterochromatic DNA has only added to its “darkness”, as sequencing of these DNA regions has been only recently achieved. Despite such obstacles, research on heterochromatin blossomed over the past decades. Success in this area benefitted from efforts of Sergio Pimpinelli and colleagues who made landmark discoveries and promoted the growth of an international community of researchers. They discovered complexities of heterochromatin, demonstrating that a key component, Heterochromatin Protein 1a (HP1a), uses multiple mechanisms to associate with chromosomes and has positive and negative effects on gene expression, depending on the chromosome context. In addition, they updated the work of Carl Waddington using molecular tools that revealed how environmental stress promotes genome change due to transposable element movement. Collectively, their research and that of many others in the field have shined a bright light on the dark side of the genome and helped reveal many mysteries of heterochromatin.

## 1. Heterochromatin: From the Dark to the Light

In the early 1900s, geneticists, such as Muller, Painter, and Schulze, described two types of chromosomal regions in the eukaryotic nucleus [1,2]. One type was electron dense, remained condensed during interphase, and possessed relatively few genes. The second type was electron poor, decondensed during interphase, and possessed many genes. Condensed chromosomal regions were referred to as “empty” and “inert.” Heitz describe them as “deviant behaving parts of single chromosomes” and labeled these regions “heterochromatin” [3]. Today, we understand that heterochromatin is enriched at centric and telomeric regions and is required for many chromosome functions including nuclear compartmentalization, genome stability, and chromosome integrity [4,5,6,7]. While heterochromatin is mostly a transcripitionally repressive chromatin environment, it possesses a small collection of genes that require heterochromatin for expression [5,8,9,10].

Heterochromatin is a mosaic of simple DNA repeats and a patchwork of complete and incomplete transposable elements (TEs) that cluster in “transposon graveyards” [11,12,13,14,15]. Nearly all eukaryotic genomes carry heterochromatic regions with copious numbers of TEs [14]. In *Drosophila*, TEs represent nearly 20% of the genome, with at least 30% corresponding to full length, active elements [16]. In humans, TEs represent nearly 45% of the genome, but are mostly inactive [16]. Transposons deploy self-encoded integrases, endonucleases, and transposases to move around genomes. As they move, they carry with them promoters, transcription factor binding sites, and polyadenylation signals [12,15,16,17,18]. Consequently, TEs have the capacity to alter transcriptional regulation, promote ectopic recombination, and generate chromosome rearrangements within host genomes. In some cases, TEs have been co-opted to perform critical functions [12], exemplified by *Drosophila* telomeres comprised by the HeT-A, TART, and TARHE TEs [17,19,20]. As such, TEs are components of heterochromatin that confer innovation to genomes, while posing a threat to genome stability.

Much of our knowledge about the composition, structure, function, and plasticity of heterochromatin comes from studies in *Drosophila*. Formative discoveries were brought to light during International Conferences on *Drosophila* heterochromatin that began through the vision of Sergio Pimpinelli and Maurizio Gatti, who saw a need to gather scientists with a shared fascination for the dark side of the genome [21,22,23]. The first conference was held in May of 1990 in Bari, Italy. Occuring on a biennial basis thereafter, this conference has attracted *Drosophila* and non-*Drosophila* researchers alike, as properties of *Drosophila* heterochromatin are conserved among species. Here, we highlight research of Sergio Pimpinelli and his colleagues, as first described at these conferences. For decades, studies from this group have expanded our understanding of the functions of Heterochromatin Protein 1a (HP1a) [24,25,26,27], a key component of heterochromatin, and mechanisms by which transposon movement has been linked to environmental stress. Together, these studies have provided a more complete picture of genome organization, transcriptional regulation, and evolutionary change.

## 2. Expanding Functions of Heterochromatin Protein 1a

**Figure 1:**
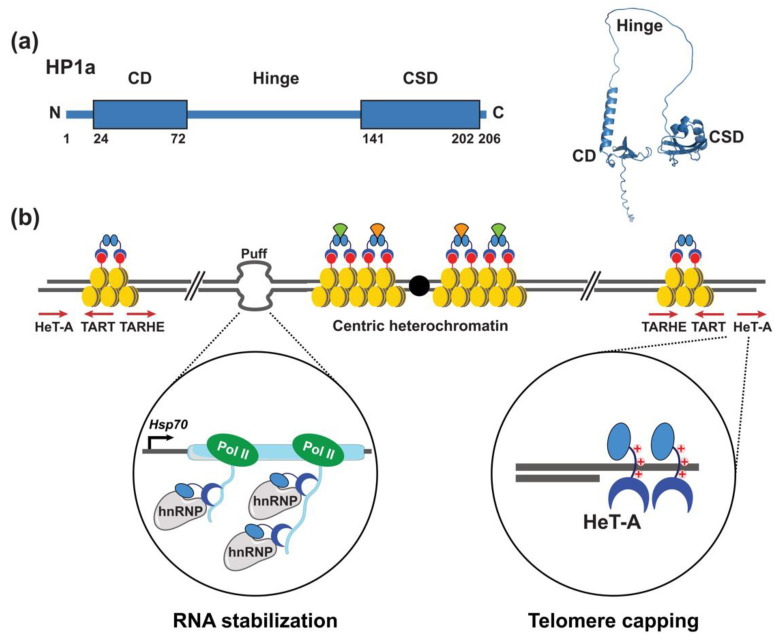
HP1a uses multiple mechanisms to associate with chromosomes. (**a**) Left: HP1a has a conserved domain structure consisting of a chromodomain (CD) and chromoshadow domain (CSD) that flank a flexible hinge region. Right: Ribbon diagram of model full length HP1a as predicted by AlphaFold 2.0 [43]. (**b**) The CD of HP1a binds histone H3 lyine 9 di- and tri-methyl (H3K9Me2/3; red circles) and silences gene expression. (**b**) HP1a associates at centric, telomeric and eucrhomatic regions using different mechanisms. At centric regions, HP1a associates with nucleosomes (yellow) through the histone H3K9me2/3 epigenetic mark [28]. Dimerization of the HP1a CSD forms a platform for the interaction of chromatin modifying factors, many of which contain a PxVxL pentapeptide (orange and light green shapes). HP1a binds to euchromatic sites that “puff” as a result of high levels of expression of heat shock and developmentally regulated genes. At puffs, HP1a binds nascent mRNAs through the CD, and associates with hnRNPs to stabilize and/or promote proper processing of mRNAs (enlarged circle on the left). HP1a binds directly to telomeric DNA sequences through ts positively charged amino acids in the hinge to cap chromosome ends (enlarged circle on the right). Icons used in this figure were modified from bioicons (bioicons.com); https://smart.servier.com/ (accessed on 20 December 2021) is licensed under CC-BY 3.0 Unported https://creativecommons.org/licenses/by/3.0/ (accessed on 20 December 2021) and, DBCLS https://togotv.dbcls.jp/en/pics.html (accessed on 20 December 2021) is licensed under CC-BY 4.0 Unported https://creativecommons.org/licenses/by/4.0/ (accessed on 20 December 2021).

Heterochromatin Protein 1a (HP1a) is a member of a conserved family of chromatin proteins that share a domain structure consisting of an N-terminal chromodomain (CD) followed by a less conserved hinge region (H) and a C-terminal chromoshadow domain (CSD), which can homodimerize [24,27] (Figure 1a). First demonstrated by immunohistochemical studies of HP1a association with giant *Drosophila* salivary gland polytene chromosomes [25,26], HP1 proteins broadly distribute throughout metazoan genomes, localizing to centric and telomeric chromosomal regions, as well as some euchromatic sites. HP1a binds chromosomes via recognition of the CD with histone H3 di- and tri-methylated lysine 9 (H3K9me2/3), an epigenetic mark generated by the conserved histone methyltransferase SU(VAR)3-9 [28] (Figure 1b). Interactions between HP1a and SU(VAR)3-9 propagate heterochromatin along the chromosome, generating domains of silent chromatin [28,29,30,31,32]. The CSDs of HP1 dimers form a platform for interaction with chromatin proteins that possess the pentapeptide PxVxL (x = any amino acid) or a variant of that sequence [33,34,35,36,37] (Figure 1b). CSD interaction partners include the histone demethylase dKDM4A [38], which co-localizes with HP1a within centric heterochromatin and plays a role in repair of heterochromatic DNA damage [35,39,40]. Other CSD interaction partners play roles in the formation of ectopic heterochromatin formed at transgenes arrays inserted in euchromatin [41,42]. For example, an interaction between HP1? and the PxVxL peptide of chromatin assembly factor 1 (CAF-1) at transgene repeats causes chromatin compaction and transcriptional represssion of the repeats [42].

In *Drosophila*, loss of HP1a causes telomeric fusions, consistent with a role in capping chromosome ends [44]. Telomeric association of HP1a was assumed to occur through an interaction with H3K9me2/3, as this epigenetic mark is enriched at telomeres. However, Pimpinelli and co-workers showed that a mutant version of HP1a that lacked the ability to bind H3K9me2/3 remained associated at telomeres [45]. This surprising result was followed by chemical cross-linking that showed HP1a associated with telomeric HeT-A sequences. Subsequent gel-shift experiments demonstrated that positively charged amino acid sequences with the HP1a hinge were responsible for binding HeT-A sequences [45] (Figure 1b). In addition to this direct DNA binding, the chromodomain contributes to transcriptional silencing of TEs by binding H3K9me2/3. These discoveries expanded the repertoire of mechanisms by which HP1a associates with chromosomes and laid the foundation for future research centered on HP1-DNA interactions. Indeed, HP1a was subsequently found to bind a DNA repeat in the 5′ untranslated region of the gypsy-like ZAM TE, with evidence that DNA binding was independent of the chromodomain [46]. In addition, HP1-DNA interactions play genome-wide roles in chromatin compaction and spacial organization of the genome within the nucleus [24,47,48,49,50,51,52,53].

**Figure 2:**
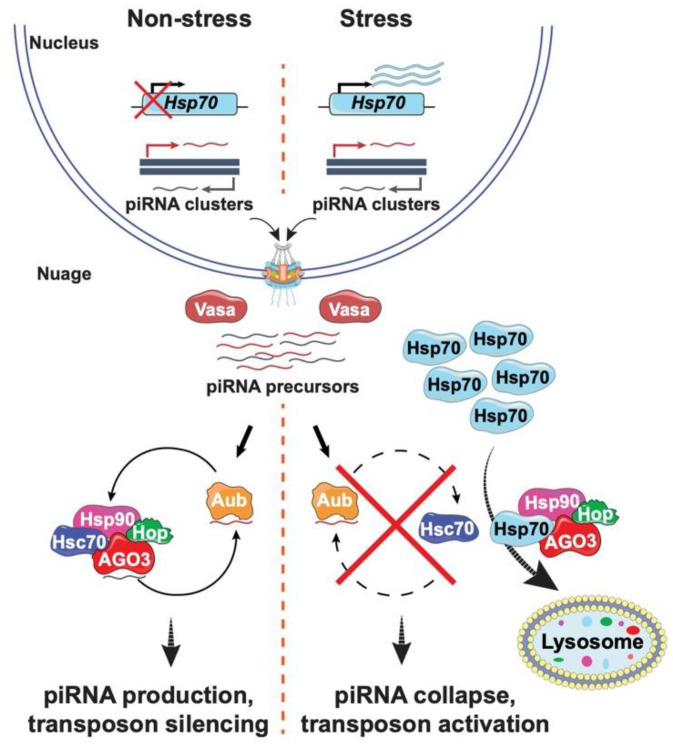
Transposon silencing collapses under stress conditions. Left: piRNA production occurs in the absence of stress. Precursor piRNAs are synthesized in the nucleus, transported into the nuage, where they are loaded onto the PIWI proteins. Aubergine (Aub) binds antisense piRNAs (red) that cleave transposon mRNAs to generate sense piRNAs (black) that are loaded onto a second PIWI protein AGO3. This cycle continues, thereby amplifying levels of piRNAs. Right: Under stress conditions, transcription of *Hsp70* increases, increasing levels of HSP70, a protein that interacts with the HSP90/83 co-chaperone complex and relocalizes Hsp90, Hop, and AGO3 from the nuage to the lysosome for degradation. As a result, stress causes collapase of piRNA synthesis and promotes transposon activation. Icons used in this figure were modified from bioicons (bioicons.com); https://smart.servier.com/ (accessed on 20 December 2021) is licensed under CC-BY 3.0 Unported https://creativecommons.org/licenses/by/3.0/ and, DBCLS https://togotv.dbcls.jp/en/pics.html (accessed on 20 December 2021) is licensed under CC-BY 4.0 Unported https://creativecommons.org/licenses/by/4.0/.

Motivated by observations that the distribution of HP1a was not restricted to centric and telomeric regions, Pimpinelli and colleagues used high resolution confocal microscopy to map HP1a binding sites within the euchromatic arms of larval salivary gland polytene chromosomes [54]. These and further studies established that at several euchromatic sites, HP1a bound to chromosome “puffs” (decondensed chromatin) in developmental and heat shock genes (Figure 1b) [55], which are caused by high levels of transcription. Strikingly, the formation of chromosome puffs was not dependent on HP1a; however, high levels *Heat shock protein 70* (*Hsp70*) gene expression required HP1a association. Treatment of polytene chromosomes with RNase eliminated HP1a association with puffs and a mutant version of HP1a with disrupted chromodomain function also eliminated binding, suggesting that HP1a binds RNA through the chromodomain [55] (Figure 2).

To develop a comprehensive understanding of the RNAs bound by HP1a, Pimpinelli and colleagues carried out RNA immunopreciptation experiments (46). Over 100 euchromatic transcripts were found in association with HP1a. Genes encoding these transcripts required HP1a for expression. HP1a was also found to associate with heterogeneous nuclear ribonucleoproteins (hnRNPs) at euchromatic loci, as well as centric heterochromatic repeats [56]. Taken together, these findings demonstrate that HP1a positively regulates euchromatic gene expression, in addition to its well-known role of silencing genes in heterochromatin [57]. Further, these studies established a new mode of chromosome association for HP1a that involved RNA. Indeed, subsequent studies have shown centric and telomeric localization of the human orthologue of HP1a depend on association with major satellite repeat RNAs and the TElomeric Repeat-containing RNAs (TERRAs) transcribed from pericentric and telomeric regions, respectively [49].

## 3. Connecting Transposon Regulation to Environmental Stress

Heterochromatin possesses a patchwork of TEs. Movement of these elements is tightly regulated to ensure genome stability. The primary germline regulator of TE propagation is the PIWI (P-element induced wimpy testis) interacting (pi) RNA pathway (Figure 2) [58,59,60,61]. This pathway is essential for female fertility and gamete quality [62,63,64]. Defense against TE movement depends upon a continuous production of small RNAs and the formation of piRNA-induced silencing complexes (piRSCs), built with members of the PIWI clade of the Argonaute family, including Piwi, Aubergine (Aub) and Argonaute 3 (AGO3) [60,65]. piRNAs are produced from long piRNA precursors generated by transcription of piRNA clusters that correspond to genomic regions of transposon relics, i.e., TE graveyards [66]. Precursor RNAs then travel to the perinuclear nuage for processing and amplification, as this subcellular location is enriched for piRNA machinery such as the PIWI clade endonucleases Aub and AGO3, the RNA helicase Vasa, and Tudor domain proteins (Figure 2) [61,67]. Vasa facilitates precursor transport to the nuage, where Aub bound antisense piRNAs direct cleavage of transposon mRNAs. Consequent cleavage products are displaced by Vasa and the 3′ fragments are loaded onto AGO3. AGO3 bound piRNAs target and cleave complementary antisense transposon transcripts, with consequent cleavage products subsequently re-loaded onto Aub. piRNAs produced by this ping-pong cycle are also loaded onto Piwi, allowing Piwi nuclear entry for direct repression of target transposons through H3K9me3 modification [58,60].

The regulation of transposons by piRNAs depends on the molecular chaperone Heat Shock Protein (HSP)90, a connection first made by Pimpinelli, Bozzetti and colleagues [61]. Their breakthrough came from studies of a hypomorphic allele of *Drosophila hsp90/83*, called *hsp83^scratch^*, a mutant that reduces piRNA biogenesis, increases TE transcript levels, and causes defects in germline development [61]. Notably, the reduced fertility of *hsp83^scratch^* mutants is coupled with increased levels of morphological defects in the offspring. Molecular analyses revealed that at least one of these mutant offspring was caused by a *de novo* TE insertion [61], foreshadowing subsequent studies showing that heat shock related chaperones contribute to RNA-induced silencing [68]. Indeed, HSP90 forms active complexes with piRNAs and Piwi [69] and the Hsc70/Hsp90 chaperone machinery contributes to loading of small RNAs onto Argonaute proteins. Taken together, these data reveal that HSP90 and co-chaperones are integral components of the germline piRNA pathway.

Establishment that HSP90 has a central role in germline transposon silencing shed new light on the old question of how animals adapt to environmental stress. In the 1940s, Conrad Waddington proposed that environmental stress uncovers cryptic genetic variants pre-existing in genomes that are naturally buffered by other genes to prevent minor changes in developmental programs [70]. Waddington predicted that the constancy of a wild-type phenotype is built by natural selection, which leads to canalization of these developmental processes. Even so, Waddington noted that when development occurs under extreme environmental conditions, pre-existing variants can be revealed, selected for, and assimilated in subsequent generations [70]. To test this hypothesis, Waddington exposed a natural wild population of *Drosophila* to daily heat shocks during pupal development. He found that such treatment generated morphological changes in offspring, changes that could be selected for during subsequent generations of heat exposure and that became stabilized once offspring were grown under normal conditions. Working from the assumption that heat was non-mutagenic, Waddington concluded that an abnormal pre-existing phenotype was newly canalization, such that the altered phenotype remained upon restoration of the growth conditions [71].

The concept of canalization and buffering was revitalized in the early 2000s, as genetic and pharmacological inhibition of Hsp90/83 uncovered morphological changes in nearly every tissue of the fly, identifying Hsp90/83 as a factor capable of acting as a capacitor or buffer of cryptic genetic variation [72]. Yet the advances of Pimpinell, Bozzetti and colleagues challenged these conclusions, as Hsp90 is required for suppression of TEs movement. Motivated by these their new data, Pimpinelli and colleagues revisited the Waddington experiment [73]. To this end, two newly isolated wild *Drosophila* stains were subjected to daily heat shocks during pupal development and four morphological mutant phenotypes were selected over several generations and shown to produce heritable phenotypes in the absence of heat. Applying molecular tools, the genetic basis of the mutations was identified. All four mutants resulted from either transposon insertion or genomic deletion, genetic changes that were absent in the parental stains. This molecular return to Waddington’s experiments establishes a clear connection between TE movement and heat stress, implying that the major mechanism underlying genetic assimilation of heat induced phenotypes is co-selection of *de novo* germline mutations, not selection of pre-existing mutations [74,75]. Consistent with this interpretation, previous studies had shown that most spontaneous mutations in natural *Drosophila* populations result from transposon insertions [74,75].

Yet, a role for HSP90 in piRNA production did not fully explain the heat sensitivity of the piRNA pathway, as this chaperone is produced under non-stress conditions. This knowledge gap was again filled by the Pimpinelli group, with the discovery that Hsp70 has a central role in altering TE activity after heat stress [76]. Heat stress induces production of high levels of Hsp70. As a result, Hsp70 interacts with the Hsp90/83 co-chaperone complex and relocalizes this complex from the nuage to the lysosome, causing degradation of the piRNA machinery (Figure 2). Consequently, piRNA biogenesis collapses. These data of Pimpinelli and colleagues suggest that while Hsp70 protects animal survival against environmental stress, elevated levels of this chaperone also increase the frequency of mutation in the germline. Such mutations might provide innovations for adaptability and evolution that promote survival of subsequent generations. Critically, the wide-spread nature of TEs among diverse organisms, coupled with the conservation of components of the piRNA pathway, reinforce the role of TEs in driving genome change and evolution.

## 4. Coalescence of a Heterochromatin Community

Over the past several decades, research in the areas of heterochromatin structure and function, TE biology, and genome evolution have taken a prominent position in genome research. Pimpinelli and coworkers have sparked many of the key advancements that led to this notoriety. Through their own studies and their organization of conferences, an international network of researchers coalesced, and the free exchange of ideas and tools allowed brightness to shine on the dark side of the genome. Without pre-existing bias, these scientists have uncovered exceptions to rules that led to the development of new paradigms. Excitement remains high, as there are likely to be many more secrets buried within heterochromatin that remain to be discovered.

## Data Availability

Not applicable.
